# Assessing opportunities for physical activity in the built environment of children: interrelation between kernel density and neighborhood scale

**DOI:** 10.1186/s12942-015-0027-3

**Published:** 2015-12-22

**Authors:** Christoph Buck, Thomas Kneib, Tobias Tkaczick, Kenn Konstabel, Iris Pigeot

**Affiliations:** Leibniz Institute for Prevention Research and Epidemiology, BIPS, Bremen, Germany; Faculty of Economic Sciences, University of Göttingen, Göttingen, Germany; Institute of Geography, University of Bremen, Bremen, Germany; National Institute for Health Development, Tallinn, Estonia; Institute of Psychology, University of Tartu, Tartu, Estonia; Faculty of Mathematics and Computer Science, University of Bremen, Bremen, Germany

**Keywords:** Active living, Adaptive bandwidth, IDEFICS study, Moveability, Spatial scale, Urban neighborhood, Walkability

## Abstract

**Background:**

Built environment studies provide broad evidence that urban characteristics influence physical activity (PA). However, findings are still difficult to compare, due to inconsistent measures assessing urban point characteristics and varying definitions of spatial scale. Both were found to influence the strength of the association between the built environment and PA.

**Methods:**

We simultaneously evaluated the effect of kernel approaches and network-distances to investigate the association between urban characteristics and physical activity depending on spatial scale and intensity measure. We assessed urban measures of point characteristics such as intersections, public transit stations, and public open spaces in ego-centered network-dependent neighborhoods based on geographical data of one German study region of the IDEFICS study. We calculated point intensities using the simple intensity and kernel approaches based on fixed bandwidths, cross-validated bandwidths including isotropic and anisotropic kernel functions and considering adaptive bandwidths that adjust for residential density. We distinguished six network-distances from 500 m up to 2 km to calculate each intensity measure. A log-gamma regression model was used to investigate the effect of each urban measure on moderate-to-vigorous physical activity (MVPA) of 400 2- to 9.9-year old children who participated in the IDEFICS study. Models were stratified by sex and age groups, i.e. pre-school children (2 to ${<}6$  years) and school children (6–9.9 years), and were adjusted for age, body mass index (BMI), education and safety concerns of parents, season and valid weartime of accelerometers.

**Results:**

Association between intensity measures and MVPA strongly differed by network-distance, with stronger effects found for larger network-distances. Simple intensity revealed smaller effect estimates and smaller goodness-of-fit compared to kernel approaches. Smallest variation in effect estimates over network-distances was found for kernel intensity measures based on isotropic and anisotropic cross-validated bandwidth selection.

**Conclusion:**

We found a strong variation in the association between the built environment and PA of children based on the choice of intensity measure and network-distance. Kernel intensity measures provided stable results over various scales and improved the assessment compared to the simple intensity measure. Considering different spatial scales and kernel intensity methods might reduce methodological limitations in assessing opportunities for PA in the built environment.

**Electronic supplementary material:**

The online version of this article (doi:10.1186/s12942-015-0027-3) contains supplementary material, which is available to authorized users.

## Background

There is broad evidence that environmental opportunities in the urban neighborhood can positively affect health outcomes such as obesity, hypertension, and other cardiometabolic risk factors by promoting physical activity (PA) of residents [1–4]. However, findings regarding the association between the built environment and PA are still difficult to compare or pool [1, 4, 5]. In particular, differences in the results are induced by varying definitions of neighborhood scale and an inconsistent use of urban measures to assess the built environment [3–7]. In the last decade, the assessment of built environment characteristics that identified walkable and health promoting neighborhoods shifted more and more to the application of objective measures based on spatial data using a variety of methods [6–9]. However, the definition of the geographical context, i.e. the neighborhood of individuals, differs throughout many studies [1, 6, 10].

An important step to assess the built environment is the definition of the spatial context, i.e. the neighborhood scale, in which the built environment is assumed to affect the individual [1, 4, 10–13]. Most of the studies that investigated the built environment relied on pre-defined administrative areas [1], though these are known to induce bias and spatial misclassification of neighborhoods. First, spatial movement and behavior of an individual is not bound to artificially defined areas. Assigning spatial information in one district to residents that are more attracted to adjacent districts might lead to a misclassification of urban measures, which was previously described as the container effect [14]. Second, the size and proportion of e.g. census districts or zip codes induce differing results due to changes in spatial scales, which is commonly known as the modifiable areal unit problem (MAUP) [13, 15].

Ego-centered neighborhoods [10, 16] that assess built environment characteristics based on a pre-defined distance around the place of residence can avoid the MAUP as well as the container effect and seem to be suitable to assess urban measures [8, 9, 15]. However, studies that used ego-centered neighborhoods did not use the same distance to determine individual network-dependent neighborhoods in which built environment measures were assessed. Since the neighborhood distance influences study results, discrepancies in the use of spatial scale hinder a comparison of the findings reported in the literature [5, 11]. Recent reviews of built environment factors that focussed on either PA [6], cardiometabolic risk factors [1], or obesity [4] found multiple ranges applied for ego-centered neighborhoods and discussed contradictory results and varying definitions of spatial scale. Brownson et al. [6], for instance, found studies using ego-centered neighborhoods that ranged from 500 m up to 3.2 km (up to 2 miles) and that were mostly determined by assuming a 10 min walk. Casey et al. [4] as well as Leal and Chaix [1] found some studies that used ego-centered neighborhoods with euclidian or network-based distances which, however, ranged from 400 m to 5 km [4] or from 100 m to 4.8 km [1]. Only few of these studies considered the effect of neighborhood scale on their results [4].

In addition, studies often used a fixed neighborhood distance for the assessment of the built environment in general, though the spatial context of the association between the built environment and PA might differ by age groups, sex, or other individual level characteristics [12]. Older adults, for example, or disabled persons might interact with a quite small neighborhood compared to younger and healthier adults [13]. Boone-Heinonen et al. [17] evaluated urban measures using buffer distances from 1 to 8.05 km and found the strongest association between urban characteristics and PA in different network-distances depending on the considered urban characteristic. Overall, a broad definition of the spatial context in which point characteristics should be assessed to capture the exposure of the built environment induces an uncertainty that adds to the methodological gap in the recent literature. This gap was particularly discussed as the Uncertain Geographic Context Problem (UGCoP) by Kwan [13].

Another difficulty in comparing studies of the built environment is caused by the choice of the geostatistical method [6, 14] and the fact that different measurements were found to affect the association between the built environment and PA [18]. The appropriate assessment of the built environment depends on a thorough modeling of point characteristics. The simple intensity approach that calculates urban measures as number per area of the chosen neighborhood is commonly used, but it is based on the assumption of a non-varying mean of point characteristics in the study area [19]. Since urban environments show strong spatial variation in built environment characteristics, an inhomogeneous intensity measure is recommended [14, 19, 20]. The inhomogeneous intensity assesses urban point characteristics by a smoothed intensity surface in terms of a weighted average that is calculated independently from a specific delineation of neighborhoods. In a previous study, we found an association between the built environment and PA levels in children on a macro-level. Here, urban measures were calculated based on a kernel approach within administrative areas, where the kernel intensity of point characteristics improved the assessment compared to the simple intensity [19]. In further research, we also used the kernel intensity on a micro-level and found it to be a useful method to asses built environment characteristics in ego-centered neighborhoods compared to the simple intensity [21].

However, the kernel approach is mainly determined by the choice of the bandwidth [20]. Commonly, a fixed bandwidth is used to estimate the inhomogeneous point intensity, though the choice of the optimal bandwidth as a smoothing parameter is difficult due to a trade-off between bias and variance of the kernel estimator. A data-driven choice of the bandwidth based on cross-validation might improve the intensity measure compared to a pre-defined value [22].

Using bandwidths adaptive to a varying background information of the study area may enhance the kernel intensity measure in urban environments [23, 24]. A fixed bandwidth does not account for the underlying residential density that directly influences, for example, the availability of public open spaces or intersections. A kernel intensity measure based on a bandwidth adaptive to the residential density might be able to identify and quantify true hot spots that reflect the availability or density of point characteristics adjusted for space and population [23]. Shi [24] discussed the use of an adaptive bandwidth with regard to the use of kernel intensity measures in disease mapping. Considering the inhomogeneous background that is present in a varying urban environment, an adaptive bandwidth that depends on the underlying residential density might improve the assessment of environmental exposure [23].

The present study was conducted to simultaneously evaluate two important components of built environment research. First, the simple intensity and kernel intensity measures were used to assess three point characteristics such as intersections, public transit stations, and public open spaces. In particular, cross-validated and adaptive bandwidths that depend on the underlying residential density were considered to improve kernel intensity measures. Second, the influence of the neighborhood scale on the association between built environment characteristics and habitual PA in children was investigated. Urban measures were assessed in different network-distances. Analyses of the association between urban measures and PA levels were stratified by sex and age groups. Overall the study aimed to identify the influence of varying spatial scales and sex- and age-specific neighborhoods as well as a suitable method to assess point characteristics.

## Methods

### Study data

Our analysis is based on data of the Identification and prevention of Dietary- and lifestyle-induced health EFfects In Children and infantS study (IDEFICS) [25]. The IDEFICS study is a European multicenter study that was conducted from 2006 to 2012 to investigate the etiology of lifestyle- and nutrition-related diseases and disorders in 16,228 2-to 9.9-year-old children at baseline from eight European countries [26]. We used data of 448 children that lived in one German study region, Delmenhorst, Lower Saxony, and took part in the baseline survey in 2007 and 2008. The study region Delmenhorst is about 62 km$^2$ large and had about 77,300 residents in 2008.

PA was assessed based on accelerometer devices using 15 s epochs. We considered accelerometer measurements of 448 children who wore the devices for at least three consecutive days including one weekend day with at least 8 h of valid weartime each after exclusion of intervals of at least 30 min of consecutive zeros [27]. We excluded 24 children who lived in the rural peripheral area of the municipality, since we focused on children living in an urban environment. Additionally, 24 children were excluded due to missing values in questionnaire information, which resulted in a total sample of 400 2- to 9.9-year-old children. Age- and sex-specific BMI z-scores and categories for overweight and obesity were calculated according to Cole and Lobstein [28]. Our sample included slightly more girls (n = 231, 51.6 %) than boys and 75 % were school children (n = 300) aged 6–9.9 years.

Moderate-to-vigorous physical activity (MVPA) was defined using the cut-off value of 2298 counts per minute (cpm) according to Evenson [29]. In addition we considered hours of valid weartime and the season of accelerometer measurement as confounder. Seasons of assessment were categorized to spring/summer, if the accelerometer device was worn in September 2007 and between March and May 2008, and to autumn/winter, if assessment took place between October 2007 to February 2008. Moreover, education and qualification of parents were classified according to the International Standard Classification of Education (ISCED) [30]. We collapsed ISCED-levels into three categories, i.e. low (lower secondary education and less), medium (upper and post-secondary education), and high (tertiary education). Reported safety concerns of the parents that might have restricted children’s PA were identified using two items of the parental questionnaire, ‘I restrict my child’s outdoor activities for safety reasons.’, and ‘I don’t like to let my child walk/cycle to kindergarten, pre-school or school for safety reasons.’ to which parents could agree or disagree on a four point Likert-scale. Agreement and disagreement on one of these statements was condensed to a binary variable for parental safety concerns.

### Environmental data

We collected and processed spatial data of the city of Delmenhorst based on land registry data and open source data which was described previously [19, 21]. In particular, we derived the street network utilizing data of the OpenStreetMap-Project^1^ (OSM). Spatial data of point characteristics such as the street network, i.e. intersections, public transit stations, and public open spaces, such as playgrounds, parks, and public green spaces of the German study region Delmenhorst were considered for the calculation of urban measures in different neighborhood distances. Environmental data were processed and managed in *ArcGIS 10.1*^2^.

### Neighborhood distance

**Fig. 1:**
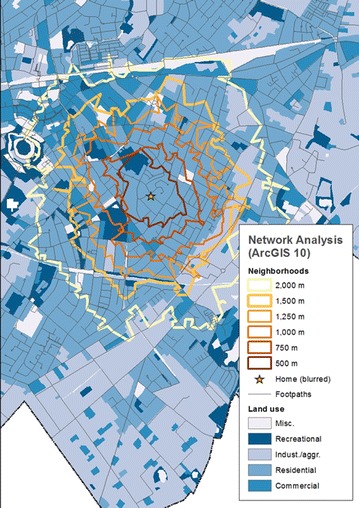
Ego-centered neighborhoods using six different network-distances based on a hypothetical place of residence in the study area Delmenhorst

For each participant, ego-centered network-dependent neighborhoods were derived based on the place of residence and the street network. To capture opportunities of the built environment in differing neighborhood contexts [12, 17], we considered six different network-distances, i.e. 500 m, 750 m, 1, 1.25, 1.5, and 2 km, for which we calculated urban measures of point characteristics. Figure 1 illustrates the extent of neighborhoods based on the six chosen network-distances from a hypothetical place of residence.

Exact address coordinates could not be used as place of residence to calculate ego-centered neighborhoods, due to data protection requirements. Therefore, we used a spatial blurring based on a Gaussian error that was inversely proportional to the underlying residential density [31, 32]. In a previous simulation, spatial blurring turned out to shift original coordinates by about 50 to 100 m in densely-populated areas and to induce only small differences of walkability measures [31].

We calculated network-dependent neighborhoods using the *network analyst* in *ArcGIS 10.1* based on the blurred coordinates and conducted spatial blurring in *R* 3.1.0 [33] using the *rnorm* function.

### Urban measures

Point characteristics of the built environment, i.e. public open spaces, intersections, and public transit stations, were modeled as Poisson point processes (PPP) [20, 22] and assessed using different intensity measures. Considering a homogeneous PPP, where it is assumed that the number of points have a constant mean over neighborhoods *A* of the study area $W\subset \mathbb {R}^2, A\subset W,$ we first assessed the availability of point characteristics by the simple intensity $\hat{\lambda }_A,$ of neighborhood *A*, i.e. as number of points $\#s_i, i=1, \ldots ,n,$ per area $\nu (A)$

$$\begin{aligned} \hat{\lambda }_A = \frac{\#{s_i \in A}}{\nu (A)}. \end{aligned}$$

In a previous study, we emphasized that the assessment of built environment characteristics in adjacent districts can be improved by kernel intensity approaches. We modeled point characteristics as an inhomogeneous PPP $\text {N}(A) \sim \text {Poi}(\Lambda _A)$ with $\Lambda _A=\int _A\;\lambda (s)\;ds$ to evaluate if kernel density approaches also improve the assessment based on ego-centered neighborhoods. Thus, we inferred the availability of point characteristics using an inhomogeneous intensity measure

$$\begin{aligned} \hat{\lambda }(s)=\frac{w_{|\Sigma |,s}}{|\Sigma |}\sum _{i=1}^n\mathbb {K}\left( |s - s_i|^T\Sigma ^{-1}|s - s_i|\right) , \end{aligned}$$

where $\Sigma$ is the covariance matrix, i.e. bandwidth, of the two-dimensional Gaussian kernel function $\mathbb {K}$ and $w_{|\Sigma |,s}$ is an edge-correction factor [19, 22, 34]. For a given neighborhood *A* the mean $\Lambda _A$ of the inhomogeneous intensity $\hat{\lambda }(s)$ for $s_i \in A$ is calculated. The choice of an adequate bandwidth is crucial when using a kernel density. A very small bandwidth will result in an undersmoothed intensity surface that manifests a large amount of single peaks, while a very large bandwidth will induce an oversmoothed intensity where less variation is visualized by the intensity surface [22, 34]. This dilemma is referred to as the well-known bias-variance trade-off [35].

First, we chose a fixed bandwidth of $\sigma _f = 500$ m of the Gaussian kernel function with regard to the isotropic case of $\Sigma = \sigma \mathbb {I}$. The fixed bandwidth $\sigma _f$ was determined by visual screening of resulting intensity surfaces based on different values for $\sigma _f$. Second, we specified a cross-validated bandwidth $\sigma _{CV}$ that was calculated by minimizing the mean square error (MSE) to choose the optimal bandwidth based on the available data of point characteristics [20, 36]. Both approaches still assume an isotropic, i.e. circular, kernel function that expands by the same distance in each direction and thus implies that point characteristics can be available in all directions. However, topographic features of the landscape can shape the built environment and can lead to a more elliptical townscape. Point characteristics might then also appear more likely according to the shape of the urban region. Modeling anisotropy is very common conducting spatial interpolation, i.e. kriging [37], and might also improve intensity measures of PPP. As a third kernel intensity measure, we therefore considered an anisotropic cross-validated bandwidth $\Sigma _{lscv}$ that allows to model an elliptical Gaussian kernel. This bandwidth is characterized by a covariance matrix $\Sigma$ that is calculated based on a least-square cross-validation [38].

Urban measures of point characteristics strongly correlate with residential density, since the spatial distribution of e.g. intersections, public transit stations, and public open spaces is particularly influenced by the number of residents that live in a certain neighborhood. Considering the spatial availability of, e.g., public open spaces, intensity measures only capture the availability per area. Hence, hot spots or clusters of public open spaces are inevitably identified where public open spaces are built according to the number of residents. Adaptive bandwidths allow to adjust the kernel intensity measure by the number of residents and smoothen the assessment of point characteristics [23, 24]. To change bandwidths depending on the underlying residential density $R_A = \#residents/km^2$, we multiplied the adjustment factor $(2000/R_A)$ with the three pilot bandwidths $\sigma _f, \sigma _{CV},$ and $\Sigma _{lscv}$ and defined adaptive bandwidths as

$$\begin{aligned} \tilde{\sigma }_f = \frac{2000}{R_A} \cdot \sigma _f = \frac{2000 \cdot km^2}{\#residents} \cdot \sigma _f \end{aligned}$$

and $\tilde{\sigma }_{CV}$ as well as $\tilde{\Sigma }_{lscv}$ accordingly. We used a pilot residential density, here 2000 residents per km$^2$, for which the pilot bandwidth remains unchanged. In areas with higher residential density, the factor reduces the pilot bandwidth and in areas with lower residential density, the factor increases the pilot bandwidth.

**Fig. 2:**
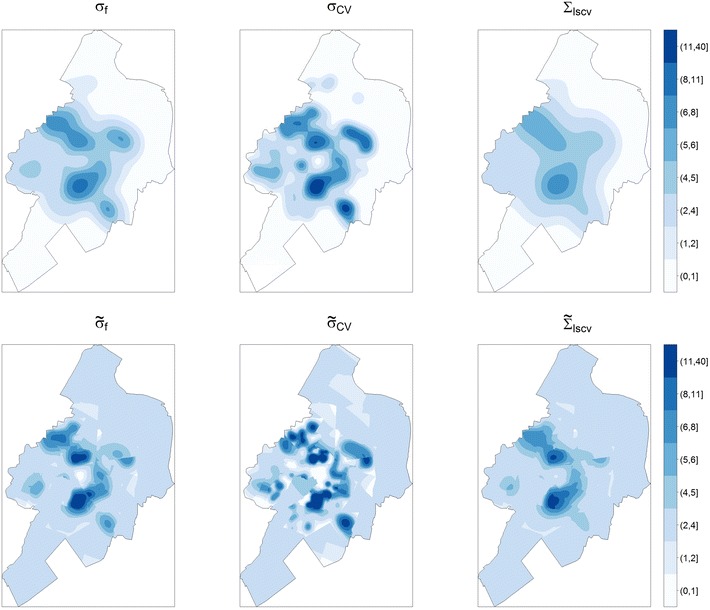
Kernel intensity surfaces based on different approaches of bandwidth selection considering a fixed (*left*), cross-validated (*middle*), or anisotropic cross-validate bandwidth (*right*), as well as a factor (*bottom*) adaptive to the underlying residential density

The seven intensity measures, i.e. the simple intensity $\hat{\lambda }_A$ and kernel intensities based on the bandwidths $\sigma _f, \tilde{\sigma }_f, \sigma _{CV}, \tilde{\sigma }_{CV}, \Sigma _{lscv},$ and $\tilde{\Sigma }_{lscv}$ were applied to calculate the mean intensity of point characteristics within six ego-centered network-dependent neighborhoods using the distances, 500, 750, 1000, 1250, 1500, and 2000 m (Fig. 1). Intensity surfaces of all six kernel intensities of public open spaces in the study area are illustrated in Fig. 2.

Urban measures were calulated using the *spatstat*-package 1-37-0 [34] in *R* 3.1.2 [33]. In detail, the MSE cross-validation was implemented using the *bw.diggle* function in *spatstat* and the least-square cross-validation was conducted using the *Hlscv* function in the *ks*-package 1.9.3 [39].

### Statistical analyses

Mean and standard deviation (SD) of individual-level variables were calculated stratified for sex and age groups. In addition, we calculated mean and SD of intensity measures based on 400 addresses considering each intensity measure and each network-distance. We calculated gamma-log-regression models to identify the effect of neighborhood distance and intensity measure on the association between urban measures and children’s habitual MVPA of children. For this purpose, we considered average minutes per day as a measure of habitual PA [27].

We first investigated the effect of individual-level variables such as age, sex, BMI z-score, ISCED level and safety issues of parents, as well as season and valid weartime of accelerometer measurements on habitual MVPA. These models are referred to as basic models in the following. Due to the skewness of the distribution and the large range of values of MVPA, we considered gamma distribution and a log-link function in our regression model. To account for differences in MVPA between boys and girls as well as pre-school children and school, which were previously identified [21], we stratified all models by age groups and by sex. As a sensitivity analysis, we also allowed for a finer age stratification to investigate the robustness of our results with respect to age strata. In addition, the performance of intensity measures and the neighborhood scale was investigated using a multi-level model that consideres daily MVPA accounting for clustering of repeated measurements nested within individuals (2679 days in 400 children).

Environmental variables, i.e. mean intensity of points per neighborhood for each type of measure based on each distance, were then separately included in the basic models. Patterns of beta estimates, *p* values, and goodness of fit based on the Akaike Information Criteria (AIC) of the corresponding model, were depicted by distance separately for different types of methods. Statistical analyses were conducted in *SAS 9.3* (SAS Institute Inc., Cary, NC, USA) and regression models were calculated using the *glimmix* procedure.

## Results

Table 1 presents descriptive statistics of the study sample. Overall MVPA levels were higher in school children (61.9 min/day) than in pre-school children (55.8 min/day) with higher values in boys (school boys 69.8, pre-school boys 58.1) than in girls (school girls 55.3, pre-school girls 52.7). Girls showed slightly higher mean values of BMI z-score compared to boys (see Table 1). Safety concerns of parents were less reported in school children (35 %) than in pre-school children (47 %) and differed between boys (38.5 %) and girls (58.1 %) only in pre-school children.

**Table 1:** Descriptive statistics of individuel-level variables in the study sample stratified by age groups and sex

Variables	Mean (SD)/N (%)		
	School children		
	All (n = 300)	Boys (n = 137)	Girls (n = 163)
MVPA (min/day)	61.9 (23.0)	69.8 (24.3)	55.3 (19.5)
Age	7(0.8).5	7.5 (0.8)	7.5 (0.8)
BMI z-score$^{\rm a}$	0.42 (1.0)	0.29 (1.0)	0.52 (1.1)
Valid weartime	11.6(1.3)	11.7 (1.4)	11.5 (1.2)
ISCED level (%)			
Low	69 (23.0)	28 (20.4)	41 (25.2)
Medium	168 (56.0)	81 (59.1)	87 (53.4)
High	63 (21.0)	28 (20.4)	35 (21.5)
Safety concerns of parents (%)			
No	194 (64.7)	87 (63.5)	107 (65.6)
Yes	106 (35.3)	50 (36.5)	56 (34.4)
Season of assessment (%)			
Autumn/winter	213 (71.0)	97 (70.8)	116 (71.2)
Spring/summer	87 (29.0)	40 (29.2)	47 (28.8)

	Pre-school children		
	All (n = 100)	Boys (n = 57)	Girls (n = 43)
MVPA (min/day)	55.8 (22.9)	58.1 (23.0)	52.7 (22.7)
Age	4.2 (0.8)	4.2 (0.8)	4.2 (0.9)
BMI z-score$^{\rm a}$	0.03 (1.1)	–0.10 (1.1)	0.19 (1.2)
Valid weartime	11.1 (1.1)	11.3 (1.1)	10.9 (1.0)
ISCED level (%)			
Low	13 (13.0)	10 (17.5)	3 (7.0)
Medium	72 (72.0)	37 (64.9)	35 (81.4)
High	15 (15)	10 (17.5)	5 (11.6)
Safety concerns of parents (%)			
No	53 (53.0)	35 (61.4)	18 (41.9)
Yes	47 (47.0)	22 (38.6)	25 (58.1)
Season of assessment (%)			
Autumn/winter	70 (70.0)	42 (73.7)	28 (65.1)
Spring/summer	30 (30.0)	15 (26.3)	15 (34.9)

^a^ According to Cole and Lobstein [28]

Table 2 shows mean and SD of intensity measures of three point characteristics depending on the considered network-distance. Mean and SD of the simple intensity were higher compared to kernel-based intensity measures with regard to all three point characteristics. Moreover, the simple intensity measure showed more pronounced differences in mean and SD between network-distances with smaller SD for larger network-distance. In contrast, kernel-based intensity measures showed more similar mean values between different types of measures and network-distances, but SD was also smaller for larger network-distances (Table 2).

**Table 2:** Mean and standard deviation (SD) of intensity of intersections, public transit stations, and public open spaces in the neighborhood of 400 children depending on network-distance and intensity measures that were used for assessment

Mean (SD)	Network-distances					
	500 m	750 m	1 km	1.25 km	1.5 km	2 km
Intensity measures						
Intersections						
Simple intensity	70.0 (26.3)	66.0 (20.5)	68.1 (16.8)	62.5 (16.4)	61.2 (15.8)	58.9 (14.5)
Fixed BW $\sigma _f$	59.8 (16.2)	59.2 (15.9)	59.4 (15.2)	58.3 (15.0)	57.7 (14.6)	56.6 (13.7)
Fixed and adaptive BW $\tilde{\sigma }_f$	62.4 (18.4)	61.6 (16.9)	61.7 (15.4)	60.6 (14.2)	60.3 (13.2)	59.5 (11.4)
MSE CV $\sigma _{CV}$	65.7 (21.4)	63.4 (18.5)	64.0 (16.2)	60.9 (15.8)	59.9 (15.2)	58.2 (14.4)
MSE CV and adaptive $\tilde{\sigma }_{CV}$	66.6 (21.8)	64.6 (18.0)	65.1 (15.8)	63.1 (14.5)	62.4 (13.6)	61.4 (12.0)
Anisotropic CV $\Sigma _{lscv}$	59.3 (16.0)	58.8 (15.7)	59.0 (15.0)	58.0 (14.8)	57.5 (14.4)	56.4 (13.5)
Anisotropic CV and adaptive $\tilde{\Sigma }_{lscv}$	62.1 (18.3)	61.3 (16.8)	61.4 (15.4)	60.4 (14.2)	60.0 (13.1)	59.2 (11.4)
Public transit stations						
Simple intensity	5.8 (3.3)	5.5 (2.4)	5.7 (2.0)	5.0 (1.7)	4.9 (1.6)	4.6 (1.3)
Fixed BW $\sigma _f$	4.6 (1.5)	4.6 (1.5)	4.6 (1.4)	4.5 (1.4)	4.5 (1.3)	4.3 (1.2)
Fixed and adaptive BW $\tilde{\sigma }_f$	4.9 (1.6)	4.8 (1.5)	4.9 (1.4)	4.8 (1.3)	4.7 (1.2)	4.7 (1.0)
MSE CV $\sigma _{CV}$	4.7 (1.6)	4.6 (1.5)	4.7 (1.5)	4.5 (1.4)	4.5 (1.3)	4.4 (1.2)
MSE CV and adaptive $\tilde{\sigma }_{CV}$	4.9 (1.7)	4.9 (1.5)	4.9 (1.4)	4.8 (1.3)	4.8 (1.2)	4.7 (1.0)
Anisotropic CV $\Sigma _{lscv}$	4.4 (1.4)	4.3 (1.4)	4.4 (1.3)	4.3 (1.3)	4.3 (1.3)	4.2 (1.1)
Anisotropic CV and adaptive $\tilde{\Sigma }_{lscv}$	4.7 (1.5)	4.6 (1.4)	4.7 (1.3)	4.6 (1.2)	4.6 (1.1)	4.5 (0.9)
Public open spaces						
Simple intensity	4.9 (4.2)	4.2 (2.8)	4.4 (2.2)	4.0 (1.6)	3.9 (1.4)	3.7 (1.1)
Fixed BW $\sigma _f$	4.1 (1.5)	4.1 (1.4)	4.1 (1.3)	4.0 (1.2)	3.9 (1.1)	3.8 (0.9)
Fixed BW and adaptive $\tilde{\sigma }_f$	4.6 (2.2)	4.4 (1.8)	4.4 (1.5)	4.3 (1.2)	4.3 (1.1)	4.2 (0.8)
MSE CV $\sigma _{CV}$	4.5 (2.2)	4.3 (1.9)	4.3 (1.6)	4.1 (1.4)	4.0 (1.3)	3.9 (1.0)
MSE CV and adaptive $\tilde{\sigma }_{CV}$	4.9 (2.9)	4.7 (2.1)	4.6 (1.7)	4.5 (1.3)	4.4 (1.1)	4.3 (0.8)
Anisotropic CV $\Sigma _{lscv}$	3.9 (1.2)	3.9 (1.2)	3.9 (1.1)	3.8 (1.0)	3.8 (1.0)	3.7 (0.9)
Anisotropic CV and adaptive $\tilde{\Sigma }_{lscv}$	4.3 (1.8)	4.3 (1.5)	4.2 (1.3)	4.2 (1.2)	4.1 (1.0)	4.1 (0.8)

*BW* bandwidth,* MSE* mean-square error,* CV* cross-validation

Results of the basic models are presented in Table 3. In school children BMI z-score was associated with MVPA ($\exp (\hat{\beta })=0.95$, *p* = 0.028), but stratified by sex this association was only found in school girls ($\exp (\hat{\beta })=0.94$, *p* = 0.031). Season and valid weartime were positively associated with MVPA in school children as well as in school boys and school girls in the stratified analyses. Safety concerns showed no significant association in school children and in the stratified samples. Effects of safety concerns were more pronounced in school girls ($\exp (\hat{\beta })=0.94$, *p* = 0.29) than in school boys ($\exp (\hat{\beta })=1.02$, *p* = 0.76). In pre-school children, age showed a significant association with MVPA in the full sample and stratified by sex. Here, children of parents with safety issues showed significantly reduced MVPA ($\exp (\hat{\beta })=0.86$, *p* = 0.044). In the stratified analyses, this result was found in pre-school girls ($\exp (\hat{\beta })=0.75$, *p* = 0.019), but not in pre-school boys ($\exp (\hat{\beta })=0.95$, *p* = 0.68).

**Table 3:** Results of the basic log-gamma regression model investigating individual-level factors on MVPA

Individual-level variables	$\exp \left( \hat{\beta }\right)$	*p* value	$\exp \left( \hat{\beta }\right)$	*p* value	$\exp \left( \hat{\beta }\right)$	*p* value
	School children					
	All (n = 300)		Boys (n = 137)		Girls (n = 163)	
	AIC = 2707.1		AIC = 1260.1		AIC = 1433.2	
Age	0.97	0.30	0.96	0.30	0.99	0.81
BMI z-score$^{\rm a}$	0.95	0.028	0.98	0.61	0.94	0.031
Valid weartime	1.04	0.022	1.05	0.041	1.02	0.39
Season (ref: winter/autumn)	1.17	0.001	1.19	0.011	1.14	0.042
Safety concerns (ref: no)	0.99	0.79	1.02	0.76	0.94	0.29
Low ISCED (ref: medium)	0.95	0.38	0.90	0.18	1.04	0.59
High ISCED (ref: medium)	1.01	0.88	1.00	0.96	1.05	0.54

	Pre-school children					
	All (n = 100)		Boys (n = 57)		Girls (n = 43)	
	AIC = 893.0		AIC = 519.5		AIC = 386.6	
Age	1.28	<0.001	1.24	0.008	1.33	0.001
BMI z-score$^{\rm a}$	1.03	0.46	1.04	0.34	1.02	0.70
Valid weartime	1.00	0.99	1.03	0.65	0.97	0.67
Season (ref: winter/autumn)	0.99	0.93	1.07	0.62	0.91	0.49
Safety concerns (ref: no)	0.86	0.044	0.95	0.68	0.75	0.019
Low ISCED (ref: medium)	1.12	0.33	1.18	0.28	1.04	0.85
High ISCED (ref: medium)	1.12	0.29	1.03	0.81	1.22	0.26

^a^According to Cole and Lobstein [28]

**Fig. 3:**
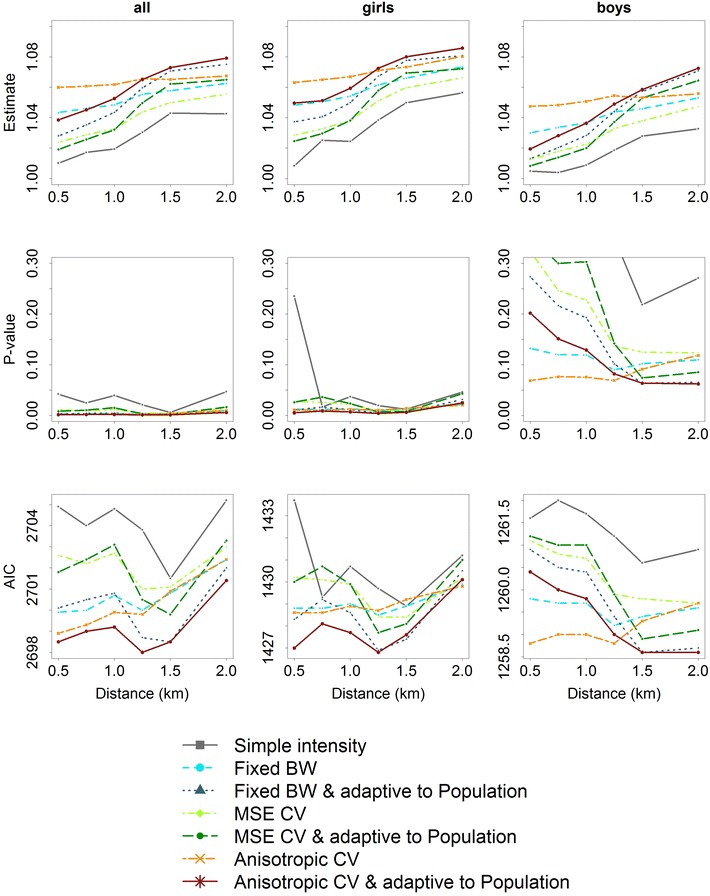
Patterns of effects (*top row*), *p* values (*middle row*), and goodness of fit (AIC) (*bottom row*) of gamma-log-regression models depending on network-distance of neighborhood and intensity measures of public open spaces in school children (*left column*), school girls (*middle column*), and school boys (*right column*)

**Fig. 4:**
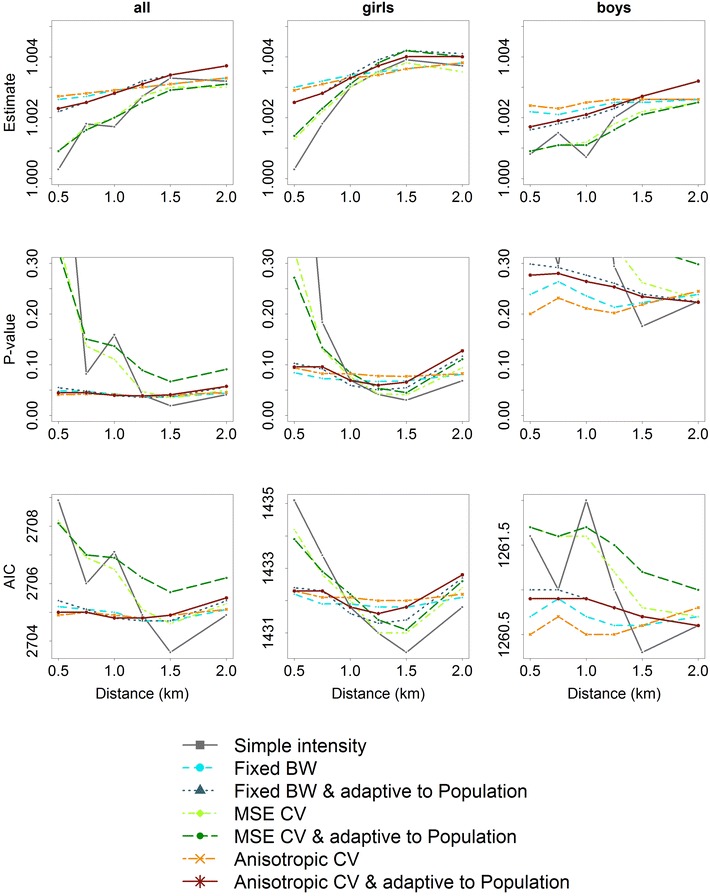
Patterns of effects (*top row*), *p* values (*middle row*), and goodness of fit (AIC) (*bottom row*) of gamma-log-regression models depending on network-distance of neighborhood and intensity measures of intersections in school children (*left column*), school girls (*middle column*), and school boys (*right column*)

**Fig. 5:**
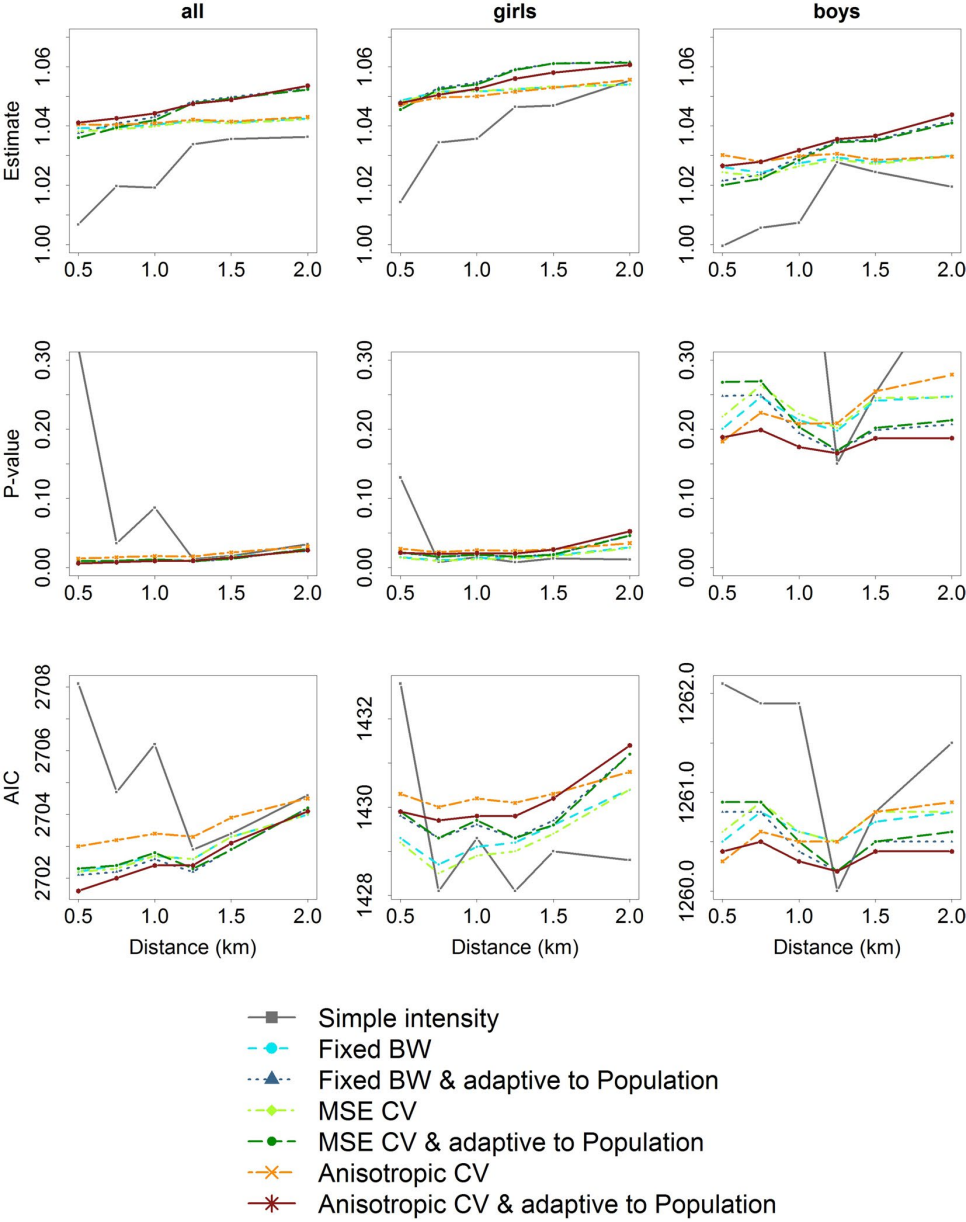
Patterns of effects (*top row*), *p* values (*middle row*), and goodness of fit (AIC) (*bottom row*) of gamma-log-regression models depending on network-distance of neighborhood and intensity measures of public transit stations in school children (*left column*), school girls (*middle column*), and school boys (*right column*)

Patterns of results of the log-gamma-regression models by network-distance and intensity measures in school children are presented in Figs. 3, 4 and 5 distinguishing between point characteristics. Additional files 1, 2 and 3 are available as supplemental material presenting the patterns of results in pre-school children.

The association between intensity of public open spaces and MVPA in school children strongly differed depending on the considered network-distance of neighborhood or intensity measure (Fig. 3). For larger network-distances the association was found to be stronger, with larger effect estimates in school girls than in school boys. *p* values showed that the association was significant in school children based on all network-distances and intensity measures. In school girls this picture only slightly changed, but in school boys *p* values strongly differed and no significant association was found. Goodness of fit only slightly differed with less variation in school boys than in school girls.

Considering the intensity measures, the simple intensity showed the smallest effect estimates and the largest values in AIC, i.e. lowest goodness of fit. In particular, effect estimates based on the anisotropic bandwidth $\Sigma _{lscv}$ showed almost no variation over network-distances followed by the adaptive version $\tilde{\Sigma }_{lscv}$. The smallest AIC, i.e. best goodness of fit, was also found for $\tilde{\Sigma }_{lscv}$ based on a network-distance of about 1250 m in school children and school girls. In school boys goodness of fit was best using a network-distance of 2 km (Fig. 3).

Figure 4 presents patterns of results of the association between intersection intensity and MVPA in school children. Again, for larger network-distances the association was found to be stronger with larger effect estimates in school girls than in school boys. *p* values also showed significant associations in school children and in school girls from a network-distance of 1250 m and more, but not in school boys. Goodness of fit slightly differed by network-distance and intensity measure considered.

Overall, intensity measures differed less for intersection intensity than with regard to public open spaces. The anisotropic bandwidth $\Sigma _{lscv}$ and the fixed bandwidth $\sigma _f$ showed the smallest variation in the effect estimates over network-distances. In all samples, goodness of fit of simple intensity measures differed ranging from the largest to the smallest AIC. The smallest AIC was observed for a network-distance of 1.5 km (Fig. 4).

Figure 5 depicts the results with regard to public transit stations which showed a similar pattern. Here, the intensity measures differed less for increasing network-distance in school children. Public transit intensity also revealed significant associations with MVPA in school children and also in school girls based on all types of measures and network-distances, but not in school boys. Three intensity measures ($\sigma _f$, $\sigma _{CV}$, $\Sigma _{lscv}$) showed almost no variation in effect estimates over different network-distances (Fig. 5).

Additional files 1 and 2 present pattern of results of the three considered point characteristics based on the sample of pre-school children. Effect estimates again differed based on the considered network-distance, but showed less variation with respect to kernel intensity measures. Particularly in pre-school boys and pre-school girls, effect estimates decreased for larger network-distance. Overall, *p* values differed strongly by intensity measure and network-distance, but no significant association was found with one exception. A significant but small association between public transit density and MVPA was found in pre-school boys based on the simple intensity using a network-distance of 500 m (Additional file 3).

## Discussion

Our results showed that the availability of public open spaces, street connectivity and the availability of public transit were positively associated with habitual PA in school children as it was previously shown [17, 21]. However, stratified results revealed the supportiveness of the built environment mainly in school girls, but not in school boys. Comparable associations of public open spaces were found in pre-school children, though street connectivity and availability of public transit showed no association with MVPA. In school children, stable results were found within a network-distance from 750 m up to 1.5 km using kernel intensity measures based on cross-validated bandwidths.

In pre-school children, effect estimates showed no association between street connectivity or availability of public transit and MVPA, but associations were found for availability of public open spaces which are similar to the association between public open spaces and MVPA in school children. Stable results were found for smaller neighborhood distances from 500 m up to 1 km, where data-driven methods again showed almost the same effect estimates over different network-distances compared to the simple intensity.

Results of the association between MVPA and urban measures based on the simple intensity, which was often used in recent publications [6, 8, 9] varied strongly by network-distance. Effect estimates of urban measures based on kernel intensity measures with cross-validated bandwidths were found to be more stable over differing network-distances. Notably, in school children, effect estimates and *p* values of urban measures based on the anisotropic cross-validated bandwidth were almost not affected by the choice of the network-distance with regard to all three point characteristics. Thus, using an anisotropic covariance matrix via cross-validation allows a more flexible modeling of the intensity and may also account for topographic features that shape the built environment and thus influence the appearance of point characteristics.

Additionally, we evaluated kernel intensity measures based on adaptive bandwidths that depend on the underlying residential density [23]. Assessing the association between point characteristics and MVPA based on an adaptive bandwidth revealed small variation in effect estimates and *p* values over network-distances from 1 to 1.5 km. Compared to the simple intensity, the anisotropic adaptive bandwidth, particularly, showed consistently larger effect estimates and smaller *p* values and showed less variation over network-distances in school children with minor disparities between school boys and school girls. The simple intensity only accounts for point characteristics within the defined neighborhood and points are either assigned or not [40]. Kernel intensity measures reduced the variance of the intensity measure compared to the simple intensity and considered point characteristics outside a defined neighborhood weighted by distance to provide a smoothed average of the availability of point characteristics within the study area. Thus, data-driven approaches might be more appropriate to assess built environment characteristics within the home neighborhood with less impact on results due to the use of different buffer distances.

In our study, goodness of fit showed only minor variation and the small differences in the AIC did not allow to identify an optimal approach or an optimal network-distance. Spielman and Yoo [12] conducted a simulation study and found that goodness of fit is not adequate to identify the optimal spatial scale in which the built environment might be assessed. For example, differences found in the spatial extent of boys and girls based on goodness of fit cannot be used to explain their spatial behavior. Thus, our choice of an appropriate intensity measure and spatial scale was based on overall performance and consistency of results. Due to the differing patterns of our results, we strongly recommend sensitivity analyses using different neighborhood scales to evaluate the consistency of the results. In a recent review, Leal and Chaix [1] emphasized the need to analyze the association between environmental exposure and health outcomes conducting more detailed analyses. Ego-centered neighborhoods should be considered and analyzed on different scales, i.e. network-distances, as to detect a possible bias in the results through sensitivity analyses and to identify comparable associations [1, 11]. Thus, our results are in line with recent discussions on the definition of the spatial context and the methodological bias [1, 10, 12, 13, 15, 40, 41].

As mentioned above, we conducted two types of sensitivity analyses (results not shown), first using a refined categorization of age groups for the stratified analyses and second considering multi-level regression to account for daily variation in the MVPA measurements. Analyses stratified by four age groups ($2\, {\rm to }\, {<}4$ , $4\, {\rm to}\, {<}6$ , $6\, {\rm to}\, {<}8$ , and $8 \,{\rm to}\, 9.9$ years) and by sex were conducted to reveal possible differences in the results between the study subgroups. Compared to the presented results, similar associations of built environment characteristics and MVPA were found and kernel intensity measures also showed more stable results compared to the simple intensity in the refined age groups. However, the small sample size in each stratum was considered too small to provide reliable results. Effect estimates and *p* values of the multi-level models differed as expected, due to the higher variability of intra-personal measurements and the larger sample size. However, performance of kernel intensity measures was similar providing more stable results over differing neighborhood scales particularly by using the anisotropic bandwidth.

Differences in the association between built environment characteristics and MVPA were identified between pre-school and school children as well as between boys and girls. Parental safety concerns were found to influence MVPA in pre-school children, especially in pre-school girls. Thus, pre-school children might experience a relatively small home neighborhood in which the availability of public open spaces or street connectivity might influence their outdoor PA that is more likely to be restricted by their parents than PA in school children. However, comparing results between pre-school and school children is limited by the small sample size for pre-school children that reduced the power of our analyses. Differences in the association of public open space intensity and street connectivity between girls and boys might be explained by including more context-specific information, e.g. sports activities in the school environment or participation in sports club outside the home neighborhood. This might be resolved in our forthcoming analyses within the framework of the I.Family study (http://www.ifamilystudy.eu) where we will employ GPS-devices.

Combined GPS- and accelerometer measurements allow to assess context-specific PA patterns [40, 42, 43] or to individualize the definition of spatial context and neighborhood scale [15] to overcome the uncertain geographic context problem [13]. In our study the association between habitual PA in children and environmental characteristics within the home neighborhood was considered, though sports activities within the school environment or destinations outside the home neighborhood might add to the overall PA [43, 44]. Results indicate that the availability of public open spaces and street connectivity within the home environment substantially contribute to overall PA of children, as it was shown in the literature [43, 45]. However, GPS-measurements allow to investigate the association between built environment characteristics and PA by generating individual activity spaces [42, 46] which do not necessarily focus on the home environment, but can also cover other environments such as the school or work environment [1]. Moreover, GPS-measurements integrated in new technologies and databases allow to combine continuous environmental variables with precise measurements of behavior and exposure on the same spatial and temporal scale [47].

Beside the methodological bias, it is important to conceptualize the spatial context considering physical or behavioral differences in the study population [1, 12, 13]. With regard to differences in PA levels and the use of the built environment, we stratified our analyses according to age groups and sex, which is suitable for a small age range. However, defining population subgroups only by simple categories such as age or sex hides multiple individual-level factors that influence PA levels and the way the built environment is perceived or used [1, 13]. In particular, self-selection factors can affect, for example, the choice of living in a walkable area, due to the preference of active travel [48] and should be considered. Similarities in the study population with regard to individual behavior, self-selection, life stages, or the perception of the environment could be addressed through latent class analyses as it was conducted by Christiansen et al. [48] and Adams et al. [49].

Some limitations in our study have to be addressed. First, results of the association might be affected by the spatial blurring that was necessary to use address coordinates, though minor effects of the spatial blurring were observed with regard to the assessment of urban measures [31]. Second, parents with medium or high educational levels were overrepresented in our study. In addition, most of the accelerometer measurements took place in autumn and winter time. Both might have also affected our results.

The major strength of our study is the use of objective measurements for the outcome on the one hand and for the environmental exposure on the other hand.

## Conclusion

Considering the differences in the results of our study that are induced by the choice of intensity measure and network-distance, we found a strong variation in the association between the built environment and PA of children induced by neighborhood scale. Moreover, differences of results depending on sex and age groups as well as point characteristics revealed the possibility for false conclusions if subgroup analyses are not conducted. Taking account of important subgroups within a study sample turned out to be crucial to investigate the association between the built environment and PA levels.

Using kernel intensity measures and particularly adaptive bandwidths provided more flexibility in modeling urban measures and improved the assessment compared to the simple intensity measure by showing stable results over various spatial scales. Thus, data-driven methods might reduce methodological limitations that typically occur when assessing opportunities for PA in the built environment.

## Additional files

10.1186/s12942-015-0027-3 Patterns of effects (*top row*),* p* values (*middle row*), and goodness of fit (AIC)(*bottom row*) of gamma-log-regression models depending on network-distance of neighborhoodand intensity measures of public open spaces in pre-school children (*left column*), pre-school girls(*middle column*), and pre-school boys (*right column*).
10.1186/s12942-015-0027-3 Patterns of effects (*top row*),* p* values (*middle row*), and goodness of fit (AIC)(*bottom row*) of gamma-log-regression models depending on network-distance of neighborhoodand intensity measures of intersections in pre-school children (*left column*), pre-school girls(*middle column*), and pre-school boys (*right column*).
10.1186/s12942-015-0027-3 Patterns of effects (*top row*),* p* values (*middle row*), and goodness of fit (AIC)(*bottom row*) of gamma-log-regression models depending on network-distance of neighborhoodand intensity measures of public transit stations in pre-school children (*left column*), pre-schoolgirls (*middle column*), and pre-school boys (*right column*).

## Footnotes

1 http://www.openstreetmap.org—Open Data Commons Open Database License (ODbL).

2 ESRI 2011. ArcGIS Desktop: Release 10.1 Redlands, CA: Environmental Systems Research Institute.
