# Supplementary figures and images for: Assessing opportunities for physical activity in the built environment of children: interrelation between kernel density and neighborhood scale
Source: Int J Health Geogr. 2015 Dec 22;14:35. doi: 10.1186/s12942-015-0027-3 (PMC4689060; doi:10.1186/s12942-015-0027-3)

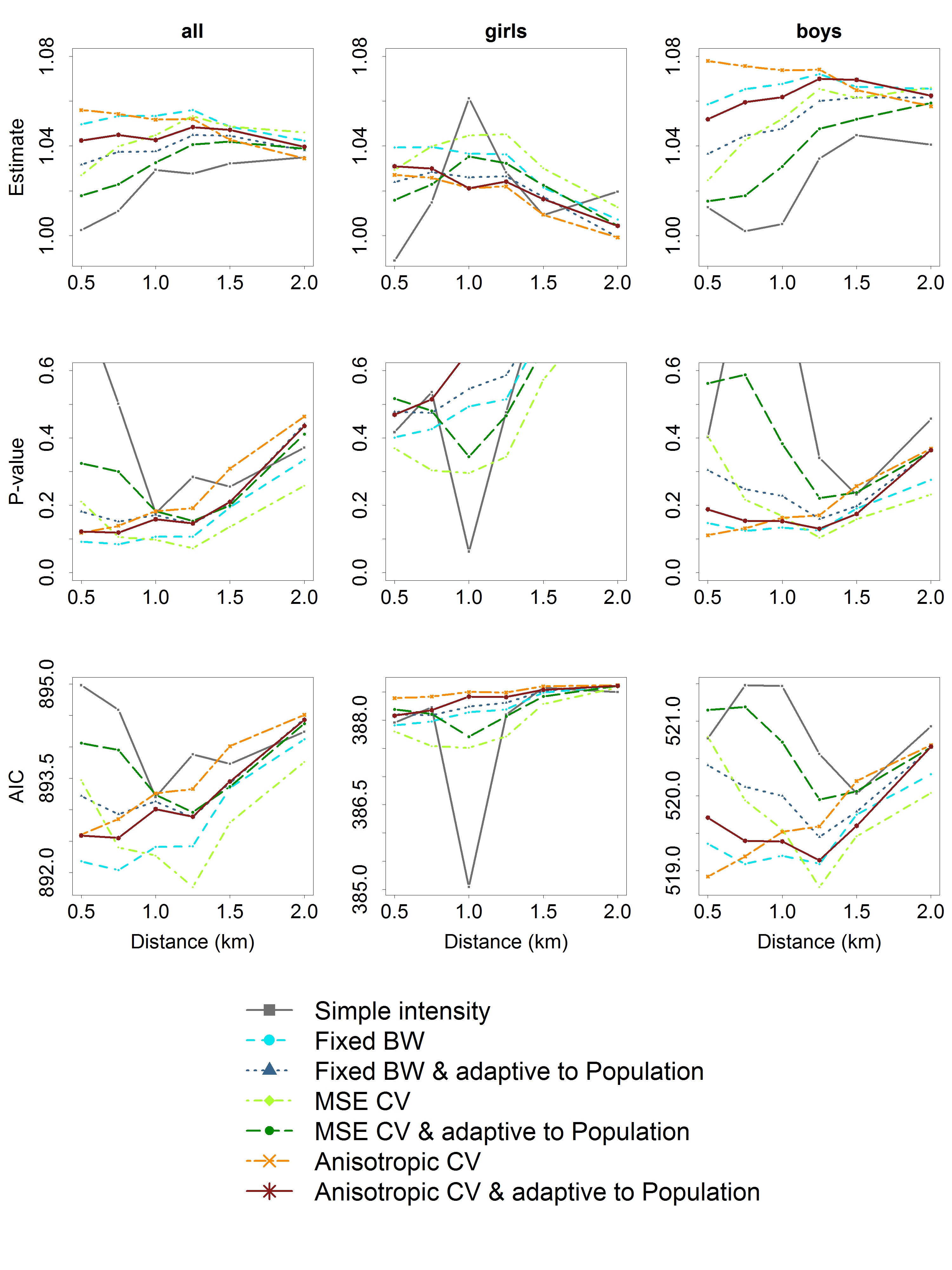

Supplement: Supplementary file 1 — 10.1186/s12942-015-0027-3 Patterns of effects (top row), p values (middle row), and goodness of fit (AIC)(bottom row) of gamma-log-regression models depending on network-distance of neighborhoodand intensity measures of public open spaces in pre-school children (left column), pre-school girls(middle column), and pre-school boys (right column). [file 12942_2015_27_MOESM1_ESM.jpg]

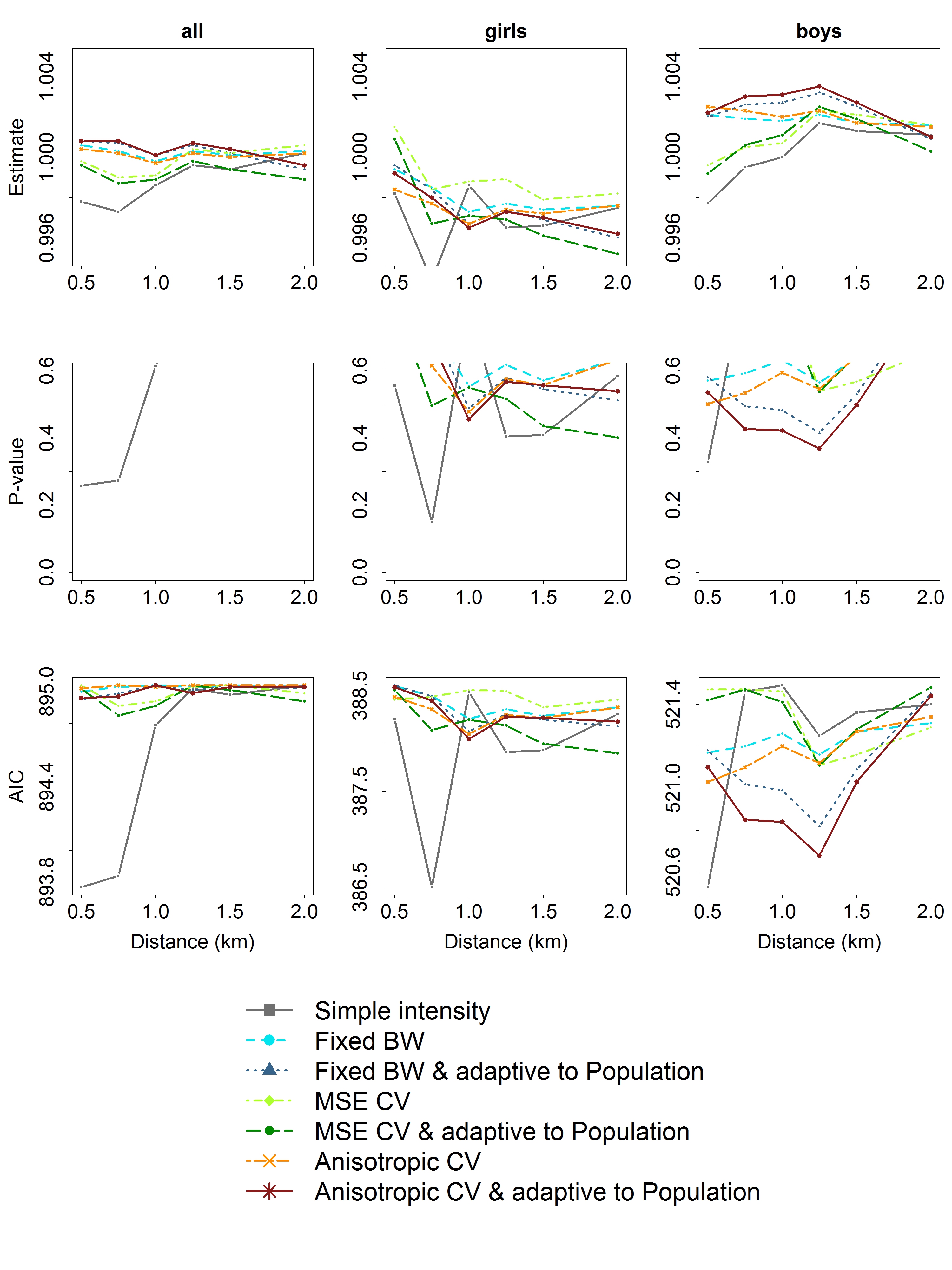

Supplement: Supplementary file 2 — 10.1186/s12942-015-0027-3 Patterns of effects (top row), p values (middle row), and goodness of fit (AIC)(bottom row) of gamma-log-regression models depending on network-distance of neighborhoodand intensity measures of intersections in pre-school children (left column), pre-school girls(middle column), and pre-school boys (right column). [file 12942_2015_27_MOESM2_ESM.jpg]

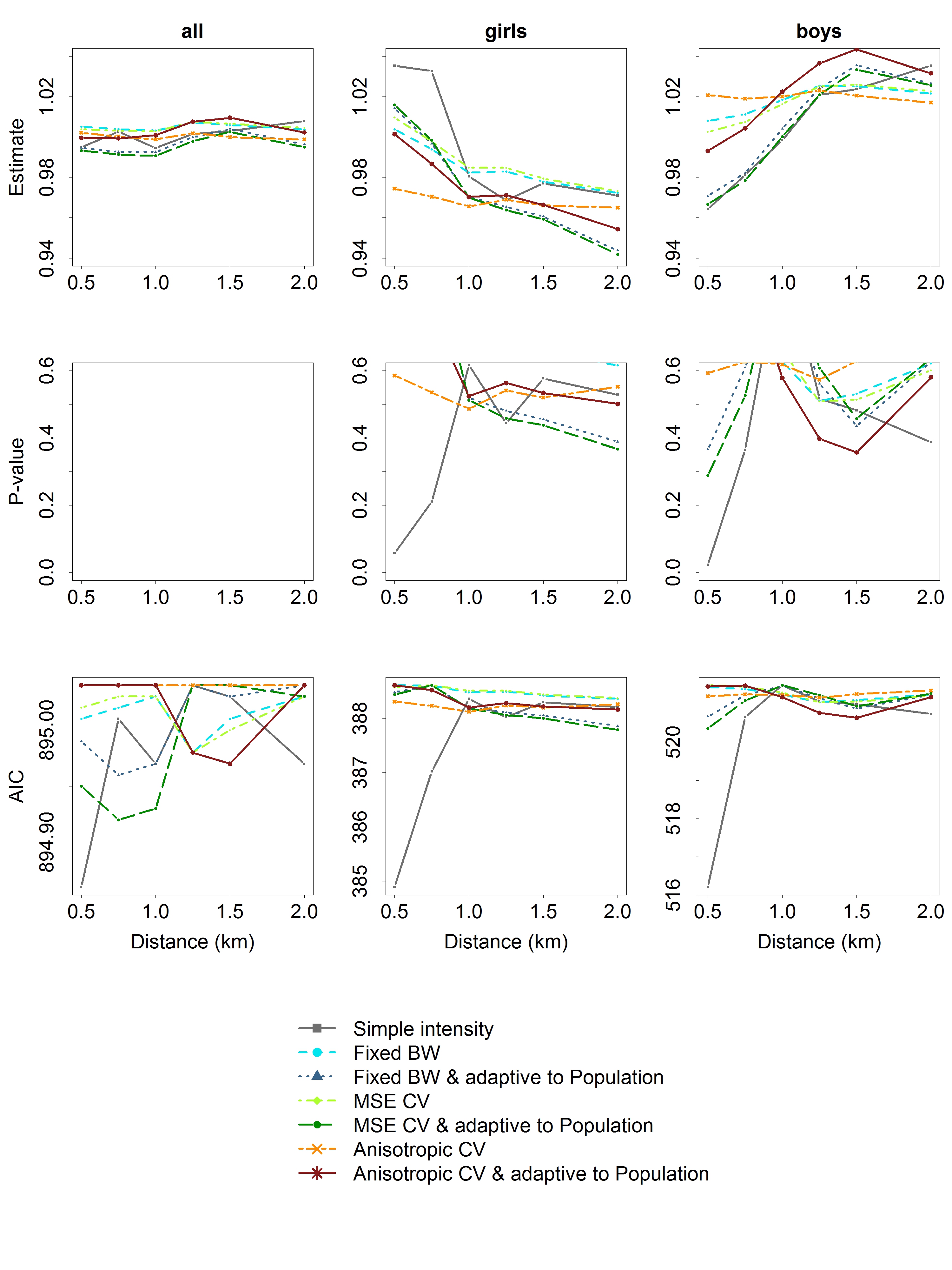

Supplement: Supplementary file 3 — 10.1186/s12942-015-0027-3 Patterns of effects (top row), p values (middle row), and goodness of fit (AIC)(bottom row) of gamma-log-regression models depending on network-distance of neighborhoodand intensity measures of public transit stations in pre-school children (left column), pre-schoolgirls (middle column), and pre-school boys (right column). [file 12942_2015_27_MOESM3_ESM.jpg]
